# Clinical and molecular characteristics, therapeutic strategies, and prognosis of non-small cell lung cancer patients harboring primary and acquired BRAF mutations

**DOI:** 10.3389/fonc.2025.1514653

**Published:** 2025-04-02

**Authors:** Xiangran Feng, Ran Zeng, Mengchen Lyu, Xiaoyan Chen, Ziwei Xu, Yue Hu, Zhiyao Bao, Xianwen Sun, Jingya Zhao, Ling Zhou, Jun Zhou, Beili Gao, Lei Dong, Yi Xiang

**Affiliations:** ^1^ Department of Respiratory and Critical Care Medicine, Ruijin Hospital, Shanghai Jiao Tong University School of Medicine, Shanghai, China; ^2^ Department of Pathology, Ruijin Hospital, Shanghai Jiao Tong University School of Medicine, Shanghai, China; ^3^ Institute of Respiratory Diseases, Shanghai Jiao Tong University School of Medicine, Shanghai, China; ^4^ Shanghai Key Laboratory of Emergency Prevention, Diagnosis, and Treatment of Respiratory Infectious Diseases, Shanghai, China

**Keywords:** BRAF, EGFR, primary, acquired, non-small cell lung cancer, co-mutation

## Abstract

**Background:**

The differences in clinical characteristics and treatment prognosis in NSCLC patients harboring primary and acquired *BRAF* mutations are still poorly understood.

**Methods:**

From Oct 2017 to Dec 2023, 10, 211 lung cancer patients at Shanghai Ruijin Hospital were reviewed. 88 primary and 15 acquired *BRAF*-mutated NSCLC patients resistant to *EGFR* TKIs were included in the study.

**Results:**

Primary *BRAF*-mutated patients preferentially occurred in the elderly (median age: 67 vs 61, p=0.015), males (53.4% vs 26.7%, p=0.056), former/current smokers (36.5% vs 6.7%, p=0.033), non-adenocarcinoma (11.4% vs 0%, P=0.351) compared to acquired *BRAF*-mutated patients. Significant differences in gender (33.3% vs 62.3%, p=0.012), smoking history (22.2% vs 43.1%, p=0.063), and adenocarcinomas (100% vs 83.6%, p=0.028) were observed between primary *BRAF*/*EGFR* co-mutated and non-co-mutated groups. While primary and acquired *BRAF*/*EGFR* co-mutated patients had similar clinical characteristics, with *EGFR* mutations being the most common coexisting oncogene (30.7% and 93.3%). The genotype of *EGFR* mutations differed, with acquired *BRAF*-mutated cases showing more complexity and a higher rate of dual *EGFR* mutations (35.7%) compared to primary cases. For primary *BRAF*/*EGFR* co-mutated patients, no matter what kinds of therapies, the *EGFR* 19del patients had a better prognosis than non-19del patients, and the first line mPFS was NR and 9.0 months (95% CI: 7.7-10.3 months) (p=0.0062), respectively. Dabrafenib and trametinib plus 3rd *EGFR* TKIs improved the prognosis of primary *BRAF*/*EGFR* non-19del co-mutated patients, achieving ORR and mPFS of 100% (3/3) and 12 months. For acquired co-mutated patients, the mPFS for 5 patients was 8.6 months (95% CI: 5.4-11.8 months). No new safety concerns and > grade 3 AEs were noted.

**Conclusion:**

Together, our study demonstrates that primary and acquired *BRAF*-mutant patients show distinct differences in some clinical and molecular characteristics, but acquired *BRAF*/*EGFR* co-mutated and primary *BRAF*/*EGFR* non-19del co-mutated patients may both respond to triple-targeted therapy.

## 1 Introduction

Lung cancer remains the leading cause of cancer mortality worldwide, owing to its high prevalence and 44.1% at advanced stage at the time of diagnosis (1, 2). The discovery of oncogenic driver alterations in non-small cell lung cancer (NSCLC) has revolutionized the treatment paradigm for patients with specific genomic alterations. Among these mutations, a significant proportion of epidermal growth factor receptor (*EGFR*) mutations are detected in 10%-15% of advanced NSCLC in Western populations and 40%-50% in Asians (3, 4). Third-generation *EGFR* tyrosine kinase inhibitors (TKIs) osimertinib has been approved as the standard treatment for *EGFR*-mutated NSCLC patients, with median progression-free survival (PFS) and overall survival (OS) reaching 18.9 months and 38.6 months, respectively (5). Previous studies have shown that patients with *EGFR* 19 deletions tend to have longer PFS and OS compared to those with L858R mutations after treatment with *EGFR* TKIs (6–8).

The V-Raf murine sarcoma viral oncogene homolog B1 (*BRAF*) is a key component in the mitogen-activated protein kinase (MAPK) pathway and has been identified as an oncogenic driver gene (9, 10). *BRAF* mutations were initially identified in melanoma with an occurrence rate of more than 60% (11). In NSCLC, *BRAF* mutations have been reported to occur in 3%-4% of Western populations (12–14) and 0.5%-2% of East Asian populations (15, 16). The *BRAF* V600E is the most common mutation, which accounts for 50%-56.8% of all *BRAF* mutations (12, 13). Notably, *BRAF* inhibitor vemurafenib or dabrafenib has achieved an objective response rate (ORR) of 33%–42% and mPFS of 5.5 to 7.3 months in *BRAF* V600E mutant NSCLC patients, respectively (17, 18). Dual-targeted *BRAF*/MEK inhibition with dabrafenib and trametinib improves therapeutic efficacy in *BRAF* V600E mutated NSCLC patients, achieving ORR and mPFS of 64% and 14.6 months (19).

With advancements in oncogenic driver gene detection technology, guidelines now recommend comprehensive genetic testing before treatment, resulting in the identification of an increasing number of co-mutations. The frequency of *EGFR* and *BRAF* co-mutations in treatment-naïve NSCLCs is reported to be 0.91% (15, 20). Due to the low frequency, little is known about the molecular biology of *BRAF*/*EGFR* co-mutations or the prognosis of *EGFR* TKIs monotherapy and *EGFR* plus *BRAF* inhibitors in *BRAF*/*EGFR* co-mutated NSCLC patients. BENEFIT study (21) showed that for advanced *EGFR*-mutated patients who received gefitinib, mPFS in *EGFR*-mutated alone vs *EGFR* with other oncogenes were 13.2 months (95%CI: 11.5–15.0) vs 4.7 months (9% CI: 1.9–9.3), which indicated that patients with concomitant oncogenes had a poor prognosis. In addition, acquired *BRAF* mutations have been identified as a resistance mechanism during *EGFR*-TKI treatment, occurring at a frequency of 1%-5% (22–24). Several studies have studied the efficacy of dabrafenib, trametinib plus osmertinib (triple-targeted therapy regimen) in these acquired *BRAF*/*BRAF* co-mutated patients, which has achieved an ORR of 58% to 80% and mPFS of 2 to 13 months (25, 26). These studies showed that triple-targeted therapy had better efficacy in acquired *BRAF*/*EGFR* co-mutated patients, but the regimen in primary *BRAF*/*EGFR* co-mutated patients has never been reported the efficacy of triple-targeted therapy regimen. Therefore, further research is required to investigate the efficacy and safety of this treatment regimen in primary and acquired *BRAF*/*EGFR* co-mutated patients.

Therefore, this study retrospectively analyzed the demographics and molecular characteristics between primary and acquired *BRAF*-mutated patients, as well as the triple-treatment regimen efficacy in these groups, and provided a new option for the treatment of primary and acquired *BRAF*-mutated NSCLC patients.

## 2 Patients and methods

### 2.1 Enrollment of patients

From Oct 2017 to Dec 2023, a total of 10, 211 lung cancer patients at Shanghai Ruijin Hospital were reviewed in our study. Primary *BRAF*-mutated patients tested *BRAF* mutation-positive before the first systematic treatment. Acquired *BRAF*-mutated patients were: 1) *BRAF* mutation-negative at baseline; 2) Detected *BRAF* mutation-positive after systematic treatment failure and subsequent rebiopsy gene testing. The inclusion criteria for patients were: (1) Histologically confirmed NSCLC; (2) *BRAF* and *EGFR* mutations detected by clinically approved sequencing platforms.

This retrospective study was approved by the Institutional Review Board of Ruijin Hospital (ID:2024-172) following the Declaration of Helsinki (revised in 2013).

### 2.2 *BRAF* and *EGFR* mutation detection

*BRAF* mutation and other gene alterations were mainly detected by amplification refractory mutation system polymerase chain reaction (ARMS-PCR) or next-generation sequencing (NGS). The ARMS molecular analysis of samples was conducted using the AmoyDx^®^ Multi-Gene Mutations Detection Kit (Amoy Diagnostics, Xiamen, China). The experiments were performed according to the manufacturer’s instructions. This kit contains 118 hotspot mutations/fusions in *EGFR*, *KRAS*, *NRAS*, *BRAF*, *ALK*, *ROS1*, *HER2*, *RET*, *MET* and *PIK3CA* genes (27). The NGS platforms used in the study encompassed various clinically approved sequencing platforms, covering panels ranging from 68 to 196 genes including the 10 driver oncogenes as above. Tissue samples were primarily used for molecular testing in most cases, while blood-based circulating tumor DNA (ctDNA) analysis was employed when tissue samples were unavailable or insufficient. All sequencing procedures followed institutional ethical guidelines and manufacturer recommendations.

### 2.3 Data collection

Baseline demographic and clinical characteristics including age, sex, pathology, smoking status, ECOG status, clinical stage, distant organ metastasis, PD-L1 tumor proportion score (TPS), molecular data, treatment regimen, efficacy, safety, and survival outcomes were extracted through a systematic review of electronic medical records. PD-L1 expression was assessed using the Dako 22C3 platform (Agilent, Santa Clara, CA, USA). Complete response (CR), partial response (PR), stable disease (SD), and progression disease (PD) were defined according to the Response Evaluation Criteria in Solid Tumors (RECIST) version 1.1. The objective response rate (ORR) was defined as CR plus PR. The progression-free survival (PFS) was the time from treatment initiation to the disease progression or death date. The overall survival (OS) was defined as the time from the diagnosis of lung cancer to death. The disease staging was determined according to the American Joint Council on Cancer (AJCC) Staging System (Version 8). Treatment-related adverse events (TRAEs) were assessed according to the Common Terminology Criteria for Adverse Events (CTCAE) version 5.0.

### 2.4 Statistical analysis

Descriptive statistics were used to characterize the patients. For categorical variables, the characteristics were described as frequency and percentages, and either the chi-square test or Fisher’s exact test was used for comparison. For continuous variables, median and interquartile were used, and the non-parametric Mann-Whitney U test was employed for comparison. Kaplan-Meier analysis and log-rank test were used to assess OS and PFS. The data were analyzed using SPSS 27.0 (IBM Corp., Armonk, New York, USA), GraphPad Prism 9.0 (GraphPad Software, San Diego, California, USA), and R software version 4.2.3 (R Foundation for Statistical Computing). The two-sided P < 0.05 was considered statistically significant. The last follow-up time was March 2024.

## 3 Results

### 3.1 The scheme of patient screening

**Figure 1:**
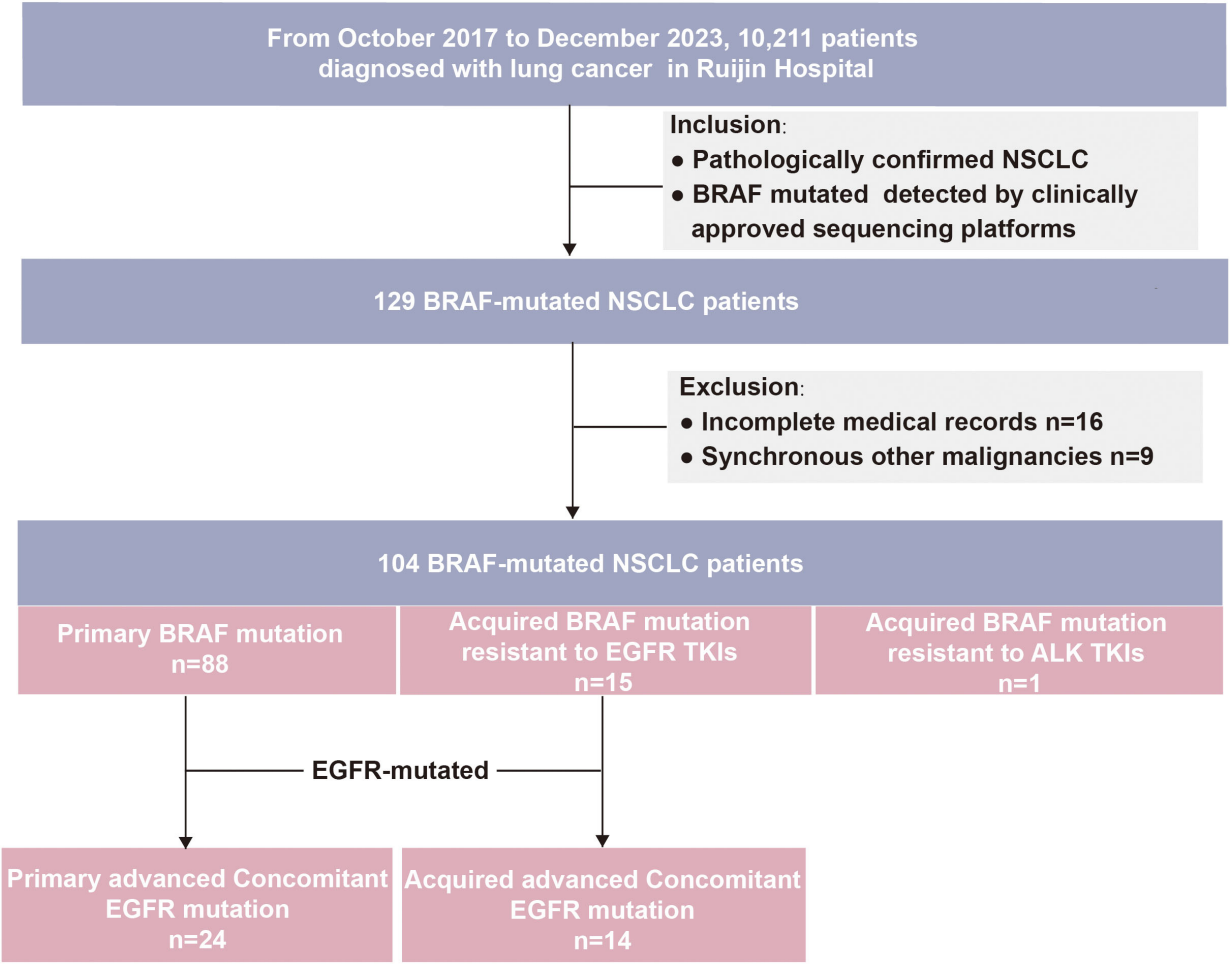
Flowchart of the patient screening.

Between Oct 2017 and Dec 2023, 129 *BRAF*-mutated lung cancer patients were identified. After excluding patients with incomplete medical records(n=16), with synchronous second primary malignancies (n=9), a total of 104 *BRAF*-mutated patients were included. In this cohort, 88 (84.6%) patients had the primary *BRAF* mutation, and 16 (15.4%) patients had the acquired *BRAF* mutation, which included one *ALK*-TKIs resistant patient and 15 *EGFR*-TKIs resistant patients. Given that this study primarily focused on patients with secondary *BRAF* mutations following resistance to *EGFR*-TKIs, subsequent research excluded patients with *ALK* TKIs resistance. Among patients with primary *BRAF* mutations, there were 23 early-stage cases (26.1%) and 65 advanced or metastatic cases (73.9%). The scheme of this study is shown in **Figure 1**.

### 3.2 Clinical characteristics of primary and acquired BRAF-mutated NSCLC patients

The median age was 67 years old (range: 28-89 years old), 32 (31.1%) had a former/current smoking history, and most were adenocarcinomas (93/103, 90.3%). All ten non-adenocarcinoma patients, including nine with squamous cell carcinoma and one with adenosquamous carcinoma, were in the primary cohort.

Primary *BRAF*-mutated patients had more elderly (median age: 67 versus 61, p=0.015), males (53.4% vs 26.7%, p=0.056), former/current smokers (36.5% vs 6.7%, p=0.033), non-adenocarcinoma (11.4% vs 0%, P=0.351) patients than the acquired *BRAF*-mutated cohort. The two groups had no differences in the distribution of *BRAF* V600E and non-V600E mutations.

The primary *BRAF*-mutated patients were divided into two subgroups, the *BRAF*/*EGFR* co-mutated cohort, and the *BRAF*/*EGFR* non-co-mutated cohort. There were significant differences in gender (males, 33.3% vs 62.3%, p=0.012), smoking history (22.2% vs 43.1%, p=0.063), and histological types (adenocarcinomas, 100% vs 83.6%, p=0.028). The proportion of PD-L1 expression TPS≥1% (41.7% vs 55.3%, p=0.411) and ≥50% (16.7% vs 23.7%, p=1.000) patients was slightly lower in primary *BRAF*/*EGFR* co-mutated patients compared to primary non-*BRAF*/*EGFR* co-mutated patients. The detailed data on clinical characteristics are summarized in **Table 1**.

**Table 1:** Clinical and molecular characteristics between primary and acquired *BRAF*-mutated NSCLC patients.

Characteristics	No. of patients (%)						
	All N=103	Primary *BRAF*-mutated N=88				Acquired *BRAF*-mutated N=15	Primary vs Acquired P value
		All N=88	*BRAF*/*EGFR* co-mutated N=27	*BRAF*/*EGFR* non-co-mutated N=61	P value		
**Age** Median (range)	67 (28-89)	67 (35-89)	64 (48-89)	68 (35-88)	0.743	61 (28-78)	**0.015**
**Gender** Male Female	51 (49.5) 52 (50.5)	47 (53.4) 41 (46.6)	9 (33.3) 18 (66.7)	38 (62.3) 23 (37.7)	**0.012**	4 (26.7) 11 (73.3)	0.056
**ECOG PS** 0-1 ≥2	91 (88.3) 12 (11.7)	79 (89.8) 9 (10.2)	26 (96.3) 1 (3.7)	53 (86.9) 8 (13.1)	0.265	12 (80.0) 3 (20.0)	0.376
**Smoking** Former/current Never Missing	32 (32.0) 68 (68.0) 3	31 (36.5) 54 (63.5) 3	6 (22.2) 21 (77.8) /	25 (43.1) 33 (56.9) 3	0.063	1 (6.7) 14 (93.3) /	**0.033**
**Histology** Adenocarcinoma Others	93 (90.3) 10 (9.7)	78 (88.6) 10 (11.4)	27 (100) 0 (0)	51 (83.6) 10 (16.4)	**0.028**	15 (100) 0 (0)	0.351
**Stage** I-IIIA IIIB-IV	23 (22.3) 80 (77.7)	23 (26.1) 65 (73.9)	3 (11.1) 24 (88.9)	20 (32.8) 41 (67.2)	**0.033**	0 (0) 15 (100)	**0.021**
***BRAF*** V600E Non-V600E	76 (73.8) 27 (26.2)	65 (73.9) 23 (26.1)	20 (74.1) 7 (25.9)	45 (73.8) 16 (26.2)	0.976	11 (73.3) 4 (26.7)	1.000
**PD-L1 TPS** <1% 1-50% ≥50% Not reported	29 (49.2) 18 (30.5) 12 (20.3) 44	24 (48.0) 15 (30.0) 11 (22.0) 38	7 (58.3) 3 (25.0) 2 (16.7) 15	17 (44.7) 12 (31.6) 9 (23.7) 23	0.755	5 (55.6) 3 (33.3) 1 (11.1) 6	0.803

ECOG, Eastern Cooperative Oncology Group; PS, performance status; PD-L1, programmed death-ligand 1; TPS, tumor proportion score.

We then analyzed the clinical characteristics of primary and acquired *BRAF*/*EGFR* co-mutated advanced patients. There were no apparent differences in gender (males, 29.2% vs 28.6%, p=1.000), smoking history (16.7% vs 7.1%, p=0.633), *BRAF* V600E (75% vs 78.6%, p=1.000) and PD-L1 TPS≥50% (10.0% vs 11.1%, p=1.000). The detailed data are shown in **Table 2**.

**Table 2:** Clinical and molecular characteristics between primary and acquired advanced *BRAF*/*EGFR* co-mutated NSCLC patients.

Characteristics	No. of patients (%)			
	All N=38	Primary *BRAF*/*EGFR* co-mutated N=24	Acquired *BRAF*/*EGFR* co-mutated N=14	P value
**Age** Median (range)	63.5 (28-89)	64 (48-89)	62 (28-78)	0.151
**Gender** Male Female	11 (28.9) 27 (71.1)	7 (29.2) 17 (70.8)	4 (28.6) 10 (71.4)	1.000
**ECOG PS** 0-1 ≥2	34 (89.5) 4 (10.5)	23 (95.8) 1 (4.2)	11 (78.6) 3 (21.4)	0.132
**Smoking** Former/current Never	5 (13.2) 33 (86.8)	4 (16.7) 20 (83.3)	1 (7.1) 13 (92.9)	0.633
***BRAF*** V600E Non-V600E	29 (76.3) 9 (23.7)	18 (75.0) 6 (25.0)	11 (78.6) 3 (21.4)	1.000
**PD-L1 TPS** <1% 1-50% ≥50% Not reported	11 (57.9) 6 (31.6) 2 (10.5) 19	6 (60.0) 3 (30.0) 1 (10.0) 14	5 (55.6) 3 (33.3) 1 (11.1) 5	1.000

ECOG, Eastern Cooperative Oncology Group; PS, performance status; PD-L1, programmed death-ligand 1; TPS, tumor proportion score.

### 3.3 Molecular characteristics of primary and acquired *BRAF*-mutated NSCLC patients

**Figure 2:**
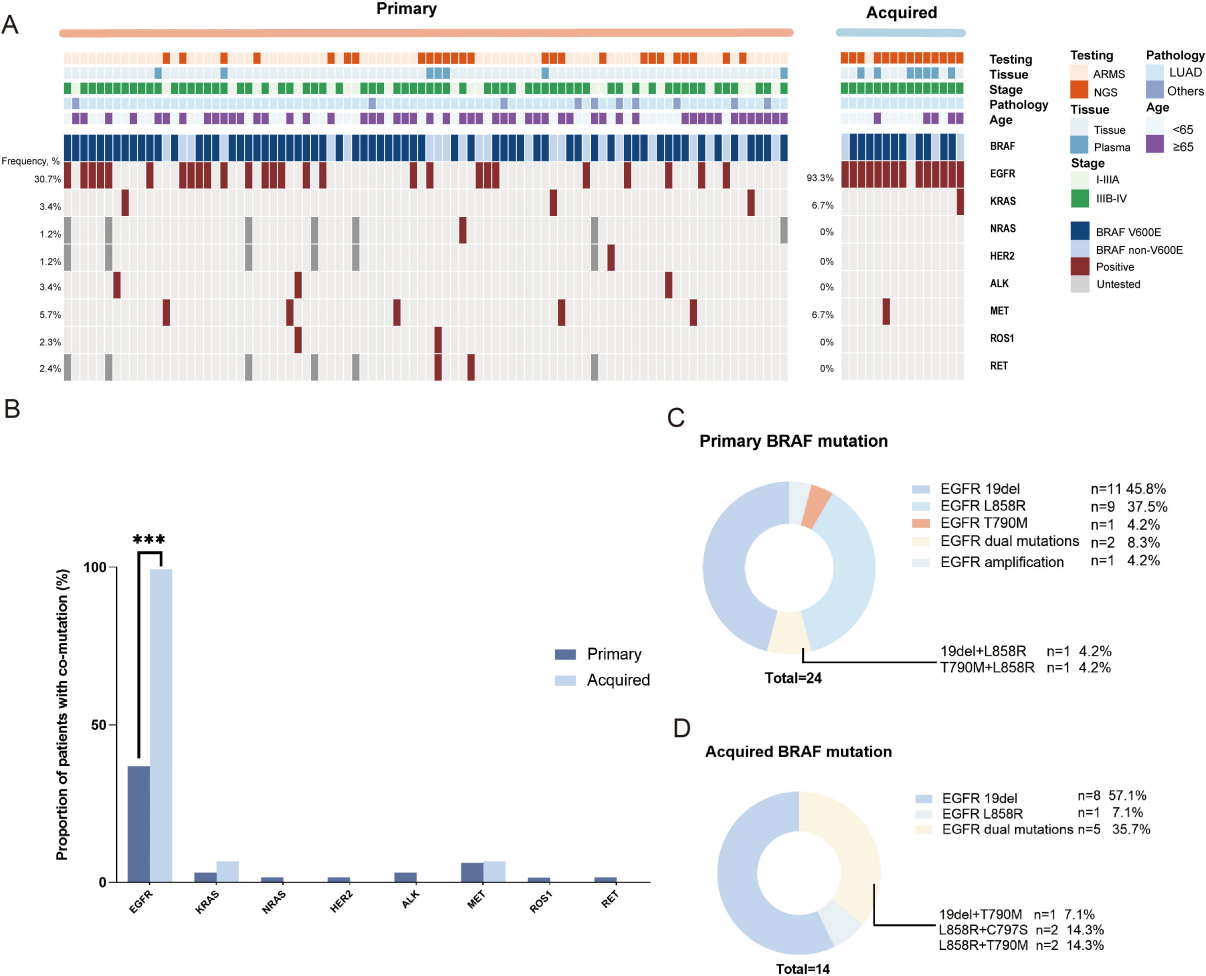
The genomic landscape of primary and acquired *BRAF* mutation resistant to *EGFR* TKIs NSCLC patients. **(A)** Oncogenic driver gene heatmap in the total patient cohort (N=103). **(B)** The bar graph of the oncogenic driver gene alterations in the advanced primary and acquired NSCLC patients. ***P < 0.001. **(C)** The type of the concomitant *EGFR* mutation in advanced primary *BRAF*-mutated NSCLC patients. **(D)** The type of the concomitant *EGFR* mutation in advanced acquired *BRAF*-mutated NSCLC patients.

We then investigated the genomic landscape of *BRAF*-mutated patients. In our cohort, among the 88 primary patients, 26 (29.5%) were tested using NGS, while 62 (70.5%) were tested using PCR. In contrast, among the acquired patients, NGS was predominantly used (14/15, 93.3%), with only one patient tested by PCR. Among patients with primary BRAF mutations, the majority of molecular analyses were performed using tissue samples (81/88, 92.0%), with only 7 cases (8.0%) analyzed via blood samples. In contrast, for patients with acquired BRAF mutations, tissue and blood samples were utilized in 8/15 (53.3%) and 7/15 (46.7%) of cases, respectively. The frequency of concomitant gene mutations was 53.4% (55/103), with 46.6% (41/88) in primary *BRAF*-mutated patients and 93.3% (14/15) in acquired *BRAF*-mutated patients (p<0.001). (**Figure 2A**). Among the primary patients, the mutation rates for *EGFR*, *KRAS*, *NRAS*, *HER2*, *ALK*, *MET*, *ROS1*, and *RET* were 30.7%, 3.4%, 1.2%, 1.2%, 3.4%, 5.7%, 2.3%, and 2.4%, respectively. Among the acquired patients, the mutation rates were 93.3%, 6.7%, 0%, 0%, 0%, 6.7%, 0%, and 0%, respectively. In advanced or metastatic NSCLC patients, 52.3% (34/65) primary *BRAF*-mutated patients and 93.3% (14/15) acquired *BRAF*-mutated patients (p=0.003) had concomitant oncogenic driver genes. The most frequently coexisting oncogenes of primary and acquired *BRAF*-mutated NSCLC patients were *EGFR* mutations (30.7% and 93.3%) (**Figure 2B**). The genotype of concomitant *EGFR* mutation differed in the two *BRAF*/*EGFR* co-mutated groups. The primary *BRAF*-mutated cohorts had *EGFR* 19del (n=11, 45.8%), *EGFR* L858R (n=9, 37.5%), *EGFR* T790M (n=1, 4.2%), *EGFR* amplification (n=1, 4.2%), *EGFR* 19del+L858R (n=1, 4.2%) and *EGFR* T790M+L858R (n=1, 4.2%). In the acquired BRAF-mutated cohorts, the genotype of EGFR mutations mainly included EGFR19 deletions (n=8, 57.1%), dual EGFR mutations (n=5, 35.7%), and L858R mutation (n=1, 7.1%). The details are shown in **Figures 2C, D**.

### 3.4 Acquired *BRAF*-mutated resistance to *EGFR* TKIs

The median follow-up time was 51.3 months (range: 11.2-153.4 months) for the 15 acquired *BRAF*-mutated patients. The acquired *BRAF*-mutated cohort included 11 (73.3%) V600E-mutated patients and 4 (26.7%) non-V600E-mutated patients. The median age detected *BRAF* mutation was 61 years old (range: 28-78). The median time from *EGFR*-TKIs treatment to *BRAF* mutation detection was 32.1 months, and the median treatment line at which *BRAF* mutation was acquired was 3 (range: 2-5). *EGFR* mutations were identified in 14 (93.3%) patients with *EGFR* 19del (n=8, 57.1%), *EGFR* L858R (n=1, 7.1%), *EGFR* L858R+C797S (n=2, 14.3%), *EGFR* L858R+T790M (n=2, 14.3%) and *EGFR* 19del+T790M (n=1, 7.1%). One patient lost the *EGFR* L858R mutation after using first-generation *EGFR* TKIs.

Five patients received dabrafenib, trametinib, and 3^rd^ generation *EGFR* TKIs (osimertinib/furmonertinib) after acquiring *BRAF* mutation. The median treatment line was 4 (range: 2-5). And the mPFS of this triple-treatment regimen was 8.6 months (95% CI: 5.4-11.8 months). Detailed information about them is provided in **Table 3**.

**Table 3:** Acquired *BRAF*-mutated NSCLC patients received triple-targeted therapy.

Case	Baseline *EGFR* mutation	Previous treatment	Mutation at resistance to Osimertinib	Treatment	Best overall response	Progression-free time
#40	*EGFR* 19del	Icotinib→Afatinib→Osimertinib	*BRAF* V600E/ *EGFR* 19del	D+T+O	PR	16 months
#59	*EGFR* 19del	Osimertinib→ Afatinib+chemo→ Almonertinib+ Savolitinib	*BRAF* V600E/ *EGFR* 19del/ *MET* amplification/ TP53	D+T+O	PD	2 months
#60	*EGFR* 19del	Osimertinib	*BRAF* V600E/ *EGFR* 19del	D+T+O	PR	>4 months
#94	*EGFR* 19del	Chemo→ Icotinib+Anlotinib→Osimertinib →Furmonertinib	*BRAF* V600E/ *EGFR* 19del	D+T+F	NA	>1 month
#95	*EGFR* 19del	Osimertinib	*BRAF* V600E/ *EGFR* 19del	D+T+F	NA	>2.4 months

D, dabrafenib; T, trametinib; O, osimertinib; F, furmonertinib, 3^rd^ *EGFR* TKI; PR, partial response; SD, stable disease; PD, progressive disease; NA, not available; Almonertinib, 3^rd^ *EGFR* TKI.

All acquired *BRAF*-mutated patients who received at least one dose of triple-targeted therapy were evaluated for safety. The most common TRAEs of any grade were pyrexia (n=3, 30%), decreased appetite (n=3, 30%), rash (n=1, 10%), fatigue (n=1, 10%), nausea (n=1, 10%), and white blood cell count decrease (n=1, 10%). There were no fatalities attributed to TRAEs (**Table 4**).

**Table 4:** Safety profile of dabrafenib and trametinib plus osimertinib.

	Grade 1-2	Grade 3	Grade 4	Grade 5
Total	14 (87.5%)	2 (12.5%)	0 (0)	0 (0)
Pyrexia	3 (18.75%)	0 (0)	0 (0)	0 (0)
Rash	2 (12.5%)	0 (0)	0 (0)	0 (0)
Fatigue	2 (12.5%)	0 (0)	0 (0)	0 (0)
Nausea	1 (6.25%)	0 (0)	0 (0)	0 (0)
Dyspnoea	1 (6.25%)	0 (0)	0 (0)	0 (0)
Peripheral edema	1 (6.25%)	0 (0)	0 (0)	0 (0)
Decreased appetite	3 (18.75%)	0 (0)	0 (0)	0 (0)
White blood cell count decrease	1 (6.25%)	0 (0)	0 (0)	0 (0)
Alanine aminotransferase increase	0 (0)	1 (6.25%)	0 (0)	0 (0)
Aspartate aminotransferase increase	0(0)	1(6.25%)	0(0)	0 (0)

### 3.5 Treatment outcome of primary BRAF-mutated patients

**Figure 3:**
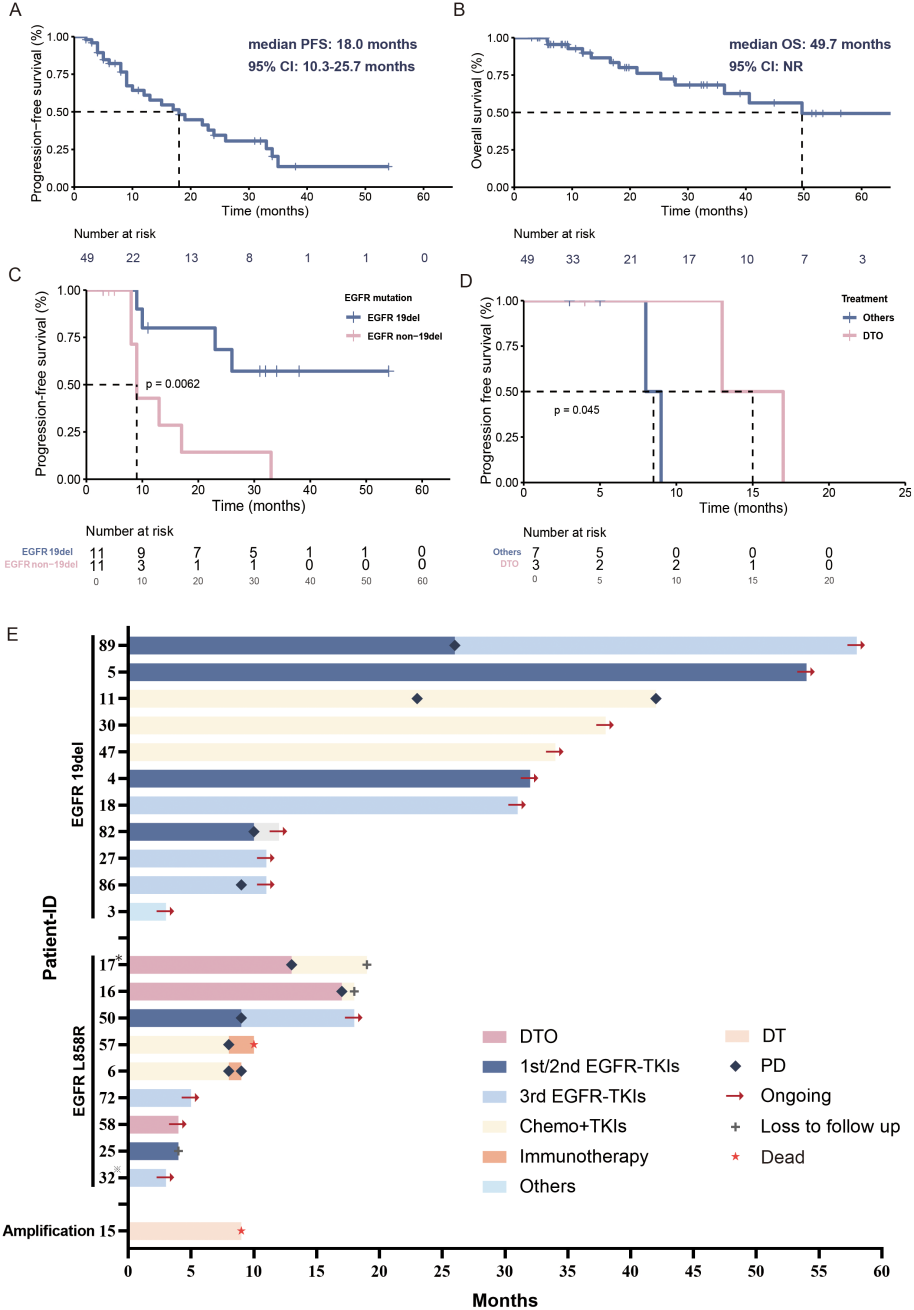
Treatment overview of primary *BRAF*-mutated patients. **(A)** Kaplan–Meier estimates of PFS of primary *BRAF*-mutated NSCLC patients. **(B)** Kaplan–Meier estimates of OS of primary *BRAF*-mutated NSCLC patients. **(C)** Kaplan–Meier estimates of PFS of primary *BRAF*/*EGFR* 19del co-mutated and *BRAF*/*EGFR* non-19del co-mutated NSCLC patients. **(D)** Kaplan–Meier estimates of PFS of primary *BRAF*/*EGFR* non-19del co-mutated NSCLC patients who received TKIs. **(E)** Swimming plot of treatment processes in primary *BRAF*/*EGFR* co-mutated NSCLC patients. ^*^Patient 17 with *EGFR* L858R+T790M mutation; ^*^Patient 32 with *EGFR* L858R+19del mutation; DTO, dabrafenib+ trametinib + osimertinib; PD, progressive disease; Others, *EGFR* TKIs/*EGFR* TKIs+chemotherapy/dabrafenib+ trametinib.

Among the 88 patients with primary *BRAF* mutations, 65 (73.9%) patients carried V600E-mutated and 23 (26.1%) carried non-V600E mutations. Of those 65 advanced or metastatic patients, 49 patients had received systematic treatment, in which the first-line regimen included targeted therapy (32/49, 65.3%), immunotherapy (11/49, 22.4%), and chemotherapy alone (2/49, 4.1%) or in combination with bevacizumab (4/49, 8.2%). Among them, 22 (44.9%) patients were primary *BRAF*/*EGFR* co-mutated, and 27 (55.1%) were non-*EGFR* co-mutated group. In the *BRAF*/*EGFR* co-mutated group, 95.5% (21/22) received targeted therapy, and 4.5% (1/22) underwent chemotherapy with bevacizumab. In the non-*EGFR* co-mutated group, first-line regimens included targeted therapy (40.7%, 11/27), immunotherapy (40.7%, 11/27), chemotherapy alone (11.1%, 3/27), and chemotherapy with bevacizumab (7.4%, 2/27). The median follow-up time was 18.0 months (range: 3.0-70.0 months). The mPFS of first-line treatments was 18.0 months (95% CI: 10.3-25.7 months), and mOS was 49.7 months (NR) (**Figures 3A, B**). Seven primary *BRAF*-mutated patients (1 with *EGFR* amplification) received dabrafenib and trametinib as the first-line treatment. The mPFS was 9.0 months (95% CI: 3.8-14.2 months). The mPFS of *BRAF* V600E and non-V600E patients was 24.0 months and 9.0 months, respectively. Regardless of the treatment line, the mPFS of 10 patients receiving dabrafenib and trametinib was 8.6 months (95% CI: 5.5–11.7 months).

We further investigated the clinical outcome of those primary *BRAF*/*EGFR* co-mutated patients (**Figure 3E**). The *BRAF*/*EGFR* 19del co-mutated patients had better PFS than non-19del (NR versus 9.0 months, 95% CI: 7.7-10.3 months, p=0.0062) (**Figure 3C**).

For these *BRAF*/*EGFR* non-19del patients, three patients (2 with *EGFR* L858R and 1 with *EGFR* T790M+L858R) received dabrafenib and trametinib plus osimertinib (triple-targeted therapy). The mPFS was 12.0 months and ORR was 100% (3/3). Other patients treated with *EGFR* TKIs (4/7, 57.1%), chemotherapy+*EGFR* TKIs (2/7, 28.6%), and dabrafenib+ trametinib (1/7, 14.3%) as the first-line regimen. Their mPFS and ORR were 8.0 months and 71.4% (5/7) (**Figure 3D**). Despite the small number of patients and short follow-up time, this triple-targeted regimen showed even better efficacy than other *EGFR* TKI-based regimes. Patient 1 received chemotherapy combined with bevacizumab, so we excluded him from the Kaplan-Meier analysis to observe the treatment prognosis of TKIs.

The adverse events associated with this triple-targeted regimen included one case of alanine aminotransferase increase (grade 3), aspartate aminotransferase increase (grade 3), rash (grade 2), and peripheral edema (grade 1); one case of dyspnoea (grade 1); and one case of pyrexia (grade 2) (**Table 4**).

### 3.6 Case of primary *BRAF*/*EGFR* co-mutated using dabrafenib and trametinib plus osimertinib with durable response

**Figure 4:**
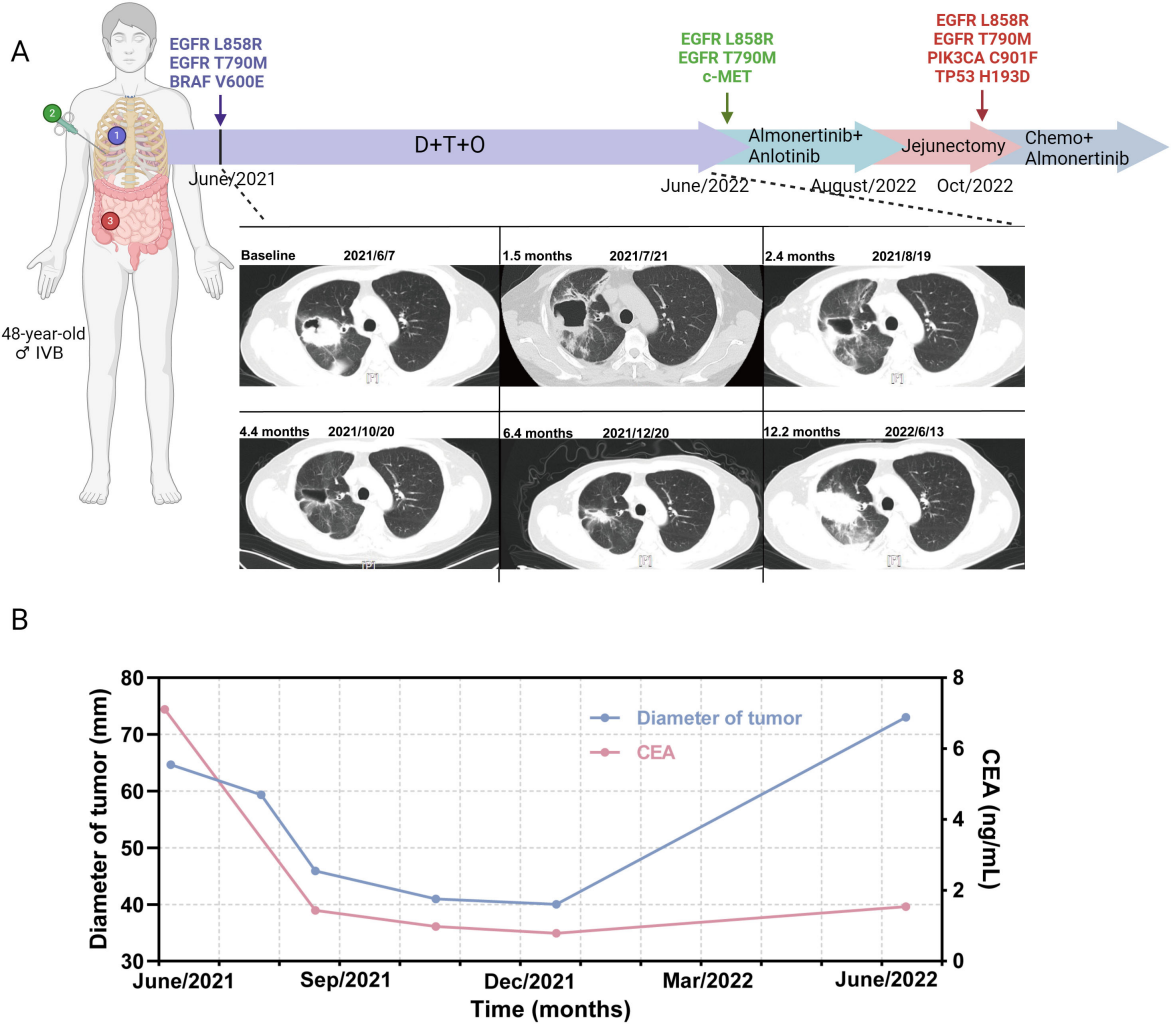
Treatment timeline and CT scans of a stage IVb adenocarcinoma patient receiving dabrafenib and trametinib plus osimertinib. **(A)** Treatment timeline and CT scans of the patient. **(B)** Tumor diameter (mm) changes and serum CEA changes during DTO treatment. CEA, carcinoembryonic antigen; D, dabrafenib; T, trametinib; O, osimertinib.

Patient 17 was a 48-year-old never-smoker male diagnosed with stage IVb lung adenocarcinoma with rib metastasis. *EGFR* L858R/T790M and *BRAF* V600E mutations were identified by ARMS PCR (**Figure 4A**). The triple treatment achieved a PFS of 12.2 months, with the best clinical efficacy being PR. The serum carcinoembryonic antigen (CEA) changes are shown in **Figure 4B**. Twelve months later, he had the progression of brain metastases. A rebiopsy was performed, and *EGFR* L858R/T790M and c-met mutations were detected. Based on the gene status alterations, treatment was changed to the third-generation *EGFR* TKI almolertinib and the multi-target tyrosine kinase inhibitor anlotinib. Two months later, due to intestinal perforation, a jejunectomy was performed. Postoperative pathology indicated metastasis of lung cancer, with genetic mutations showing *EGFR* L858R/T790M, *PIK3CA* C901F, and TP53 H193D. Additionally, liver metastasis was observed, thus subsequent treatment was changed to chemotherapy combined with almolertinib.

## 4 Discussion

To our knowledge, this is the first retrospective study to explore the clinical characteristics, molecular profiles, and treatment outcomes of primary and acquired *BRAF*-mutated patients with concomitant *EGFR* mutations. *BRAF* V600E has been identified as an oncogenic driver gene in NSCLC (18, 28), and the frequency of *EGFR* and *BRAF* co-mutation is 0.91% (20) in NSCLC patients. Moreover, multiple resistance mechanisms of *EGFR* TKIs for activating *EGFR*-mutant NSCLC patients have been elucidated, with *BRAF* being one of them (24, 29). Nowadays, several case reports and only a few retrospective studies have shown that dabrafenib and trametinib plus osimertinib might be an appropriate treatment option for acquired *BRAF*-mutated NSCLC patients. However, the clinical efficacy and safety of this triple-regimen therapy still need more clinical study. Moreover, whether this therapy regimen has better clinical efficacy on primary *BRAF*/*EGFR* co-mutated patients has never been reported.

In this study, the clinical characteristics, molecular profiles, therapeutic strategies, and prognosis of primary and acquired *BRAF*-mutated patients were analyzed. When we analyzed the clinicopathologic features, we observed several differences in the clinical features between primary and acquired *BRAF*-mutated cohorts. It has been shown that *EGFR* mutations are more frequent in non-smokers and females (30). In this study, primary *BRAF*-mutated patients were more likely to be current/former smokers, males, elderly, more complex histological types, and higher PD-L expression compared to acquired *BRAF*-mutated patients. Similar differences in clinical characteristics were observed between primary *BRAF*/*EGFR* co-mutated patients and primary *BRAF*/*EGFR* non-co-mutated patients. The primary *BRAF*/*EGFR* co-mutated patients showed similar demographics and clinical characteristics to acquired *BRAF*/*EGFR* co-mutated patients resistant to *EGFR* TKIs. Previous studies found that pre-existing T790M may exist in many TKI-naive NSCLCs, and may become the dominant tumor population as a result of drug pressure in *EGFR* TKIs resistant NSCLC patients (31). Based on this theory, primary *BRAF*/*EGFR* co-mutated patients have a higher propensity to develop dominant *BRAF*-mutated tumor clones following resistance to *EGFR* TKIs treatment. This indicated that the primary and acquired *BRAF*/*EGFR* co-mutated patients may have a common origin.

A previous large multi−center study had reported that 28 primary *BRAF*-mutated patients (11.8%, 28/238) had concomitant sensitizing *EGFR* mutations (32). In a retrospective study (33), which analyzed 53 *BRAF*-V600E mutant advanced NSCLC patients, 9 patients (9/53, 17.0%) had concomitant *EGFR* mutation, with 5 *EGFR* 19del, 3 *EGFR* L858R and 1 *EGFR* T790M. We observed that primary *BRAF*-mutated patients exhibited a high prevalence of *EGFR* mutations (27/88, 30.7%), indicating a significant co-mutation rate. This finding emphasizes the critical need to incorporate these co-mutations into first-line clinical treatment strategies. *In vitro* study (26) showed that the triple-targeted therapy of osmertinib + darafenib+ trametinib has a lower IC50 value and stronger anti-tumor effect compared with the two targeted combination regimens of osmertinib+ vemurafenib and osmertinib+ encorafenib+ cetuximab, as well as the combination of pemetrexed+ carboplatin. The tumor growth inhibition rates of these four regimens were 99.36%, 99.25%, 98.92%, and 62.83%, respectively. Hence, the triple regimen has shown good antitumor efficacy. Moreover, a Phase Ib study (34) suggests that multi-segment blockade of the RAS/RAF/ERK pathway may offer significant antitumor efficacy in patients with advanced and metastatic *KRAS* or *BRAF*-mutant non-small cell lung cancer. However, the sample size was small and the data were therefore not representative of this patient group. The effectiveness of this therapy regimen in this patient group requires further in-depth research.

Previous studies have revealed that patients with *EGFR* 19 deletions are associated with longer PFS and OS than patients with L858R after *EGFR* TKIs (6–8), and this may be due to the different phosphotyrosine patterns between the two mutations (35). Our study confirmed this result, showing that *BRAF* concomitant *EGFR* 19del patients had the longest PFS. Even with concomitant *BRAF* mutations, patients with *EGFR* 19del mutations exhibited better prognoses compared to those with non-19del mutations. In real-world practice, first-line treatment for *BRAF*/*EGFR* 19del mutated patients predominantly involves *EGFR* TKI-based therapies without selecting *BRAF*-targeted agents. Despite the insufficient follow-up period for first-line PFS data, favorable treatment outcomes are still observed. Further investigation is needed to determine if a triple-targeted regimen would offer superior efficacy compared to current treatments. For *BRAF*/*EGFR* non-19del patients, triple-targeted therapy demonstrates better outcomes than other treatment strategies (*EGFR* TKIs, *EGFR* TKIs+ chemotherapy, or dabrafenib+ trametinib). Therefore, triple therapy might be a more promising treatment approach for *BRAF*/*EGFR* non-19del patients.

However, oncogenic driver genes were previously thought to be mutually exclusive (36). *BRAF* and *EGFR* are mutually resistant mechanisms to *EGFR* TKIs or BRAF TKIs *(* 29, 37). And *BRAF* mutations are considered a negative prognostic factor (12, 38). Nevertheless, research has shown that patients harboring both *EGFR* and *BRAF* mutations can benefit from treatment with *EGFR* TKIs and BRAF TKIs *(* 39). This indicates that these patients might have varied responses to *EGFR* TKIs and BRAF TKIs. We hypothesize that the effectiveness of these drugs may depend on the activated oncogene abundance when the tumor is driven by two distinct driver genes, detecting the abundance of *EGFR* mutations and *BRAF* mutations is crucial for optimizing the selection of TKIs in clinical practice. Additionally, liquid biopsy (40) can effectively address the issue of limited biopsy sites and tumor heterogeneity in advanced patients, and can also serve as an additional option for screening gene mutations in patients, especially for patients who are unable to tolerate tissue biopsy or have poor-quality samples. Therefore, for gene mutations with high *BRAF* mutation abundance and relatively poor prognosis *EGFR* mutations, triple therapy can be considered as a treatment option.

In addition, we compared the *EGFR* mutation genotypes between the primary and acquired *BRAF*-mutated groups. The results indicated that the genotypes of coexisting *EGFR* mutations differed, with the acquired group predominantly having a complex genotype of *EGFR* mutations and more dual *EGFR* mutations (5/15, 33.3%) compared to primary *BRAF*/*EGFR* co-mutated patients. Due to the involvement of both on-target *EGFR* kinase domain mutations and bypass pathway activations in the resistance mechanisms following *EGFR* TKI treatment (24), the presence of these two mechanisms has been rarely reported. Our study demonstrated the existence of both mechanisms with a relatively high proportion. Therefore, clinical treatment needs to address both resistance mechanisms simultaneously. In the emergence of acquired resistance to *EGFR* TKIs, *BRAF* mutations have been identified as a potential alternative mechanism (29), but the subsequent therapy reports are still limited. Our study included 15 acquired *BRAF*-mutated patients after *EGFR*-TKI treatments. Previous studies have shown dabrafenib and trametinib plus osimertinib showed substantial efficacy among these *EGFR* TKIs resistant *BRAF* V600E mutant NSCLC patients, with PFS ranging from 2 to 13 months (41). 5 patients in our study received this triple-regimen treatment after acquiring *BRAF* mutations. And the mPFS of this treatment was 16.0 months (range: 2-16 months) until July 2024. One patient achieved a PFS of 16 months, and the adverse side effects were manageable. Consistent with previous studies, these results suggest the treatment can be an appropriate option for these patients.

Our study has some limitations. The first is due to the single-center retrospective nature of this study which may introduce a selection bias. The second is the small size of *BRAF*-mutated patients due to the low prevalence of *BRAF* mutations in Asian people (0.5%-1.7%) (15, 16), which hindered the possibility of stratified analysis to some extent. Third, patients were mainly detected by the 10-genes ARMS-PCR (70.5%) rather than NGS in the baseline, so the proportion of *BRAF* V600E mutation (73.9%) in the primary *BRAF*-mutated cohort was higher than other studies (50%-56.8%) (12, 13). Additionally, the use of PCR-based methods may have introduced false-negative results, as these techniques are less comprehensive compared to NGS in detecting low-frequency or non-canonical mutations. Finally, the co-mutations analyzed in our study are mainly oncogenic driver genes, the non-driven mutations still need fully investigated. Testing for more potentially predictive and prognostic alterations is expected in future study designs.

## 4 Conclusions

Our study indicated the primary and acquired *BRAF*-mutated NSCLC patients had a high frequency of coexisting *EGFR* mutations. The primary and acquired *BRAF*/*EGFR* co-mutated patients showed similar clinical characteristics and may have a common origin. Triple-targeted therapy (dabrafenib, trametinib plus 3^rd^ *EGFR* TKIs) could be considered the preferential treatment options for acquired *BRAF*/*EGFR* co-mutated and primary *BRAF*/*EGFR* non-19del co-mutated NSCLC patients. As for the primary *BRAF*/*EGFR* 19del co-mutated patients, the preferred first-line treatments still are *EGFR* TKIs-based target therapies in real-world clinical practice. Prospective randomized controlled clinical trials or larger sample-sized real-world studies are needed to confirm the effectiveness of triple-targeted therapy in primary BRAF/EGFR 19del co-mutated patients.

## Data Availability

The original contributions presented in the study are included in the article/**Supplementary Material**. Further inquiries can be directed to the corresponding authors.
