# Supplementary material for: Clinical and molecular characteristics, therapeutic strategies, and prognosis of non-small cell lung cancer patients harboring primary and acquired BRAF mutations
Source: Front Oncol. 2025 Apr 2;15:1514653. doi: 10.3389/fonc.2025.1514653 (PMC11999832; doi:10.3389/fonc.2025.1514653)
Supplement: Supplementary file 1 [file Table1.docx]

| **Stable 1: Baseline Characteristics of BRAF V600E and Non-V600E Mutated Patients in Primary and Acquired Groups.** | | | | | | | | |
| --- | --- | --- | --- | --- | --- | --- | --- | --- |
|  |  | **Primary** | | |  | **Acquired** | | |
|  |  | ***BRAF* V600E**  **N=65** | ***BRAF* Non-V600E**  **N=23** | **P Value** |  | ***BRAF* V600E**  **N=11** | ***BRAF* Non-V600E**  **N=4** | **P Value** |
| **Age**  Median (Range) | | 66(35-89) | 67(35-89) | 0.482 |  | 59.6(28-78) | 56.8(41-71) | 0.458 |
| **Sex**  Male  Female | | 33(50.8)  32(49.2) | 14(60.9)  9(39.1) | 0.404 |  | 4(36.4)  7(63.6) | 0(0)  4(100.0) | 0.159 |
| **ECOG PS**  0-1  ≥2 | | 58(89.8)  7(10.2) | 21(91.3)  2(8.7) | 0.778 |  | 10(90.9)  1(9.1) | 2(50.0)  2(50.0) | 0.080 |
| **Smoking status**  Never  Former/current  Missing | | 41(63.1)  22(33.8)  2(3.1) | 13(56.5)  9(39.1)  1(4.3) | 0.846 |  | 10(90.9)  1(9.1)  0(0) | 4(100.0)  0(0)  0(0) | 0.533 |
| **Histology**  Adenocarcinoma  Others | | 56(86.2)  9(13.8) | 22(95.7)  1(4.3) | 0.217 |  | 12(100.0)  0(0) | 4(100.0)  0(0) | / |
| **Stage**  I-IIIA  IIIB-IV | | 15(23.1)  50(76.9) | 8(34.8)  15(65.2) | 0.416 |  | 12(100.0)  0(0) | 4(100.0)  0(0) | / |
| **PD-L1 TPS**  <1%  1-50%  ≥50%  Not reported | | 19(29.2)  11(16.0)  4(6.2)  31(47.7) | 5(21.7)  4(17.4)  7(30.4)  7(30.4) | **0.023** |  | 4(36.4)  3(27.3)  1(9.1)  3(27.3) | 1(25.0)  0(0)  0(0)  3(75.0) | 0.356 |
